# Lumazine Synthase Protein Nanoparticle-Gd(III)-DOTA Conjugate as a *T*_1_ contrast agent for high-field MRI

**DOI:** 10.1038/srep15656

**Published:** 2015-10-23

**Authors:** YoungKyu Song, Young Ji Kang, Hoesu Jung, Hansol Kim, Sebyung Kang, HyungJoon Cho

**Affiliations:** 1 Department of Biomedical Engineering, School of Life Science, Ulsan National Institute of Science and Technology (UNIST), Ulsan, South Korea; 2 Department of Biological Sciences, School of Life Science, Ulsan National Institute of Science and Technology (UNIST), Ulsan, South Korea

## Abstract

With the applications of magnetic resonance imaging (MRI) at higher magnetic fields increasing, there is demand for MRI contrast agents with improved relaxivity at higher magnetic fields. Macromolecule-based contrast agents, such as protein-based ones, are known to yield significantly higher *r*_*1*_ relaxivity at low fields, but tend to lose this merit when used as *T*_1_ contrast agents (*r*_1_*/r*_2_ = 0.5 ~ 1), with their *r*_1_ decreasing and *r*_2_ increasing as magnetic field strength increases. Here, we developed and characterized an *in vivo* applicable magnetic resonance (MR) positive contrast agent by conjugating Gd(III)-chelating agent complexes to lumazine synthase isolated from *Aquifex aeolicus* (AaLS). The *r*_*1*_ relaxivity of Gd(III)-DOTA-AaLS-R108C was 16.49 mM^−1^s^−1^ and its *r*_1_*/r*_2_ ratio was 0.52 at the magnetic field strength of 7 T. The results of 3D MR angiography demonstrated the feasibility of vasculature imaging within 2 h of intravenous injection of the agent and a significant reduction in *T*_1_ values were observed in the tumor region 7 h post-injection in the SCC-7 flank tumor model. Our findings suggest that Gd(III)-DOTA-AaLS-R108C could serve as a potential theranostic nanoplatform at high magnetic field strength.

With the applications of MRI in higher magnetic fields (>7 T) both in preclinical and clinical settings increasing, there are needs to develop MRI contrast agents with improved relaxivity at higher magnetic fields. From MRI perspective, the advantage of higher magnetic field is attractive, as it is expected to drastically increase signal to noise ratio (SNR) and resolution^1^,^2^. However, it is generally observed that spin-lattice time (*T*_*1*_) increases and converges for different tissues as the magnetic field strength increases, which reduces contrast for *T*_*1*_-weighted exams using the same imaging parameters used at conventional 1.5 or 3 T^3^,^4^. As contrast to noise ratio (CNR) is critical for the sensitivity and specificity of MRI exams, this reduction of inherent *T*_*1*_ contrast necessitates the development of efficient *in vivo* compatible positive contrast agent with good relaxivity characteristics at higher magnetic field^5^,^6^,^7^,^8^.

Protein cage nanoparticles, including ferritins, encapsulins, and virus-like particles (VLPs), have been widely used as nanoscale delivery vehicles for diagnostic and/or therapeutic reagents^9^,^10^,^11^,^12^,^13^,^14^,^15^,^16^,^17^,^18^,^19^,^20^,^21^ as they not only have well-defined structure and size, but also consist of biocompatible and biodegradable biomaterials, polypeptides^22^. The highly symmetrical and uniformly composed architecture of protein cage nanoparticles allow them to be manipulated in a highly controlled manner, resulting in the production of reproducible nanoscale multifunctional particles^9^,^10^,^11^,^12^,^13^,^14^,^15^,^16^,^17^,^18^,^19^,^20^,^21^. VLPs have been extensively studied as supramolecular templates for the conjugation of small molecule contrast agents, such as the chelated paramagnetic gadolinium ion (Gd(III)), which is frequently used as a positive contrast agent for magnetic resonance imaging (MRI)^23^,^24^,^25^,^26^,^27^.

Macromolecule (such as protein)-based contrast agents are known to yield significantly higher *r*_*1*_ relaxivity due to decreased local motion and increased correlation time^28^,^29^,^30^; however *in vivo* applicable positive contrast agents, which maintain considerably enhanced *r*_*1*_ relaxivity (>10 mM^−1^s^−1^) with an acceptable *r*_*1*_/*r*_*2*_ ratio (0.5 ~ 1) at high magnetic field (7 T), are a rarity^8^,^31^,^32^,^33^,^34^.

In the present study, we used lumazine synthase, isolated from the hyperthermophile *Aquifex aeolicus* (AaLS), as a nanoscaled template for *T*_1_ contrast conjugates as illustrated in Fig. 1A and evaluated its potential as an *in vivo* MR contrast agent at the magnetic field strength of 7 T. AaLS is composed of 60 identical subunits that form an icosahedral capsid architecture (*T* = 1 state) with respective exterior and interior diameters of 15.4 nm and 9 nm^35^. AaLS is known to catalyze the penultimate step of riboflavin biosynthesis within cells^36^. The structural robustness and functional plasticity of AaLS make it an attractive candidate for a versatile bio- and nanotechnological platform^37^,^38^,^39^,^40^,^41^,^42^,^43^,^44^. Its hollow spherical architectures have been used as templates for the encapsulation of functional proteins^37^,^43^,^44^,^45^,^46^ and the biomimetic synthesis of iron oxides^44^, as well as delivery vehicles of therapeutic and/or diagnostic reagents^32^,^34^. AaLS has also been engineered into multifunctional building blocks for fabricating uniform layer-by-layer (LbL) assemblies using non-covalent interactions between surface-displayed hexahistidine and Ni-NTA of AaLS^39^. For our application, Gd(III)-chelating agent complexes were attached at position 108 with cysteine (R108C), which is known to be exposed on the exterior surface of AaLS.

Experimentally, Gd(III)-DOTA-AaLS were characterized by mass spectroscopy (MS) to ensure that AaLS was modified with only one Gd(III)-DOTA complex per subunit. Transmission emission microscopy (TEM) was used subsequently to ensure that the cage architecture and stoichiometry of AaLS is not significantly altered in the Gd complex conjugation. Next, *r*_*1*_ and *r*_*2*_ MR relaxivities were measured at 1.4 T and 7 T with multiple Gd concentration phantoms. *In vivo* imaging experiments were performed at the magnetic field strength of 7 T using a tumor bearing mouse, before and after the injection of Gd(III)-DOTA-AaLS-R108C. Observed signal behaviors were directly compared with the corresponding results from the injection of conventional Gd-DOTA (DOTAREM) for both vasculature and tumor regions.

## Materials and Methods

### Gd(III)-DOTA-AaLS-R108C as MRI contrast agent (MRI CA)

**Figure 1:**
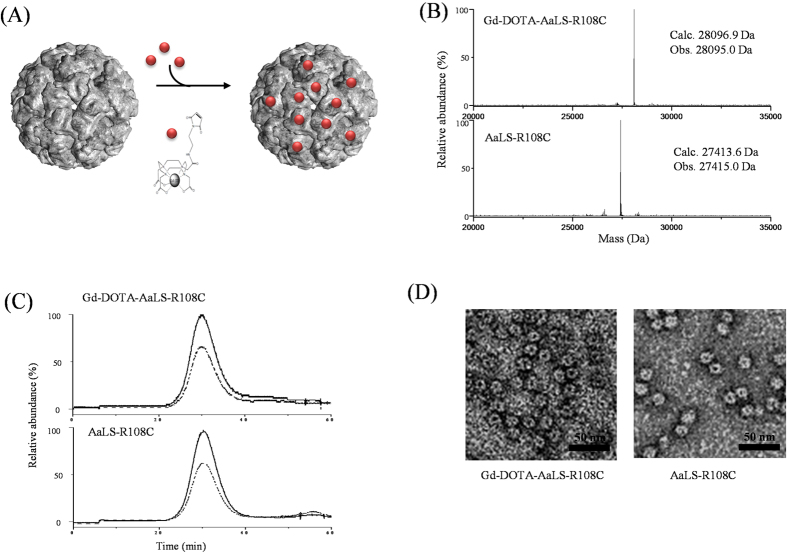
(**A**) Surface and ribbon diagram representations of AaLS-RC (PDB: 1HQK) and site-specific conjugations of Gd(III)-DOTA (red dots) to the exterior surface of AaLS-R108C. (**B**) Molecular mass measurements of dissociated subunits of untreated AaLS-R108C (bottom) and Gd(III)-DOTA-AaLS-R108C (top). Calculated and observed molecular masses are indicated. (**C**) Size exclusion profiles of untreated AaLS-R108C (bottom) and Gd(III)-DOTA-AaLS-R108C (top). (**D**) Transmission electron microscopic image of negatively stained untreated AaLS-R108C (right) and Gd(III)-DOTA-AaLS-R108C (left) with 2% uranyl acetate.

AaLS does not have sufficiently large inter-subunit spaces for Gd(III)-chelating agent complexes to freely diffuse into its interior cavity. Thus, to attach Gd(III)-chelating agent complexes in a site-specific and number controlled manner, we substituted the arginine residue at position 108 with cysteine (R108C), which is known to be exposed on the exterior surface of AaLS and readily to be used as a site for the attachment of small molecules^47^,^48^,^49^. To fully saturate the chelating agents (maleimido-monoamide-DOTA (DOTA-mal)) with Gd(III), they were incubated with an excess of Gd(III) prior to conjugation to AaLS. Subsequently, AaLS was treated with 3 mol equivalents of Gd(III)-DOTA-mal for attachment at room temperature with vigorous shaking overnight (Fig. 1A). Unreacted Gd(III)-DOTA-mal were removed by extensive dialysis with multiple changes of buffer. The extent of the conjugation was determined by subunit mass measurements using electrospray ionization time-of-flight mass spectrometry (ESI-TOF; Xevo G2 TOF).

### Cell and animal models

Squamous cell carcinoma (SCC)-7 cells were cultured in RPMI1640 medium containing 10% (v/v) fetal bovine serum and 1% (w/v) penicillin-streptomycin at 37 °C under conditions of 5% CO_2_. To generate a tumor-bearing mice model, SCC-7 (1 × 10^6^) cells in phosphate-buffered saline (PBS) were injected subcutaneously into the right flank of six-week-old female BALB/c nude mice weighing 20–25 g (Harlan Laboratories). When the tumor volume reached approximately 100 mm^3^, the mice were used for *in vivo* MR imaging. All animal studies were performed in compliance with the guidelines of the local ethics committee for animal care and use, and were approved by the Institutional Animal Care and Use Committee of Ulsan National Institute of Science and Technology.

### Relaxivity Measurements

We measured the *T*_1_ and *T*_2_ relaxation times of the Gd(III)-DOTA-AaLS under 1.4 T (Bruker Minispec MQ60 TD-NMR, 60 MHz) and 7 T (Bruker BioSpec 70/16 US, 300 MHz) magnets. At 60 MHz, the *T*_1_ relaxation times of the Gd(III)-DOTA-AaLS were measured using the inversion recovery (IR) method with the IR delay ranging from 0–20000 ms. *T*_2_ relaxation times were measured with a CPMG sequence with echo spacing (TE) of 1 ms and recycling time (TR) of 1.5 s. At 300 MHz, the *T*_1_ relaxation time was estimated using a sequence of rapid acquisition with relaxation enhancement (RARE) at variable repetition times; 10 TR values of 20–5000 ms were used with echo time (TE) of 7.6 ms. The *T*_*2*_ relaxation time was measured using a multi-slice multi-echo (MSME) method at TR of 5000 ms with 50 TE values in the range of 20–1000 ms. The proton relaxivities (*r*_*1*_ : longitudinal relaxivity and *r*_*2*_ : transverse relaxivity) were determined by measuring the *T*_*1*_ relaxation times and *T*_*2*_ relaxation times of six different Gd concentrations of Gd(III)-DOTA-AaLS-R108C (0.074, 0.037, 0.0185, 0.00925, 0.004625, and 0 mM). Both relaxivities *r*_*1*_ and *r*_*2*_ were calculated from the slope of the relaxation rate *R*_*1*_ (1/*T*_*1*_) and *R*_*2*_ (1/*T*_*2*_) as a function of concentration (mM) of contrast agent, respectively. We previously demonstrated that the relaxivities of protein cage nanoparticles conjugated with Gd(III)-chelating agent can be determined by measuring the *T*_1_ relaxation time and corresponding Gd(III) concentrations^23^. The relaxivities of Gd(III)-DOTA-AaLS-R108C were calculated using the linear relaxivity equation used in a previous study^23^,^26^. The proton relaxivities of saline solutions with untreated AaLS-R108C at identical concentrations were determined, as AaLS may also give *T*_*1*_ enhancement as well.

### *In vivo* imaging of tumor-bearing mice

All tumor-bearing mice weighing 20–25 g (tumor volume: ~100 mm^3^) were scanned using 7 T MRI (Bruker Biospec) with a transceiver RF volume coil (diameter: 40 mm) for the mouse body, maintained under anesthesia with 1.0–1.5% isoflurane. Radiofrequency power and receiver adjustments were continuously maintained for each scan. Four mice were examined to investigate the vasculature imaging capabilities and the tumor accumulation efficacy of Gd(III)-DOTA-AaLS-R108C at high field (7 T) and two mice in the control group were assessed using Gd-DOTA (DOTAREM) as a reference. Four mice were injected intravenously with 350 μl of Gd(III)-DOTA-AaLS-R108C (concentration: 17.143 mM) as a bolus into the tail vein. Also in the same manner, two mice were injected intravenously with 350 μl of DOTAREM (concentration: 17.857 mM) as a bolus into the tail vein for the direct comparisons.

For the longitudinal and direct comparison of the vasculature imaging capability and delivery efficacy of Gd(III)-DOTA-AaLS-R108C to the tumor region, three dimensional (3D) MR angiography (MRA) and *T*_*1*_-mapping were adopted at six time points (1, 2, 3, 7, 12, and 30 h) after the intravenous injection of the contrast agent at a dose of 0.3 mmol/kg. To this end, a 3D-Fast Low-Angle Shot (FLASH) and a RARE with variable repetition time TR (RAREVTR) sequence were used for a high-resolution angiogram and *T*_*1*_-map, respectively. MR parameters were set as follows: TR = 13 ms, TE = 2.1 ms, flip angle = 20°, FOV = 30 × 30 × 30 mm^3^, matrix size = 256 × 256 × 256 for 3D-FLASH; TR = 8 values in the range of 280–5000 ms, TE = 6.0 ms, field of view (FOV) = 30 × 30 mm^2^, slice thickness = 1 mm, matrix size = 128 × 128 for RAREVTR *T*_*1*_ measurements. MRA were reconstructed using maximum intensity projection (MIP) protocol with Bruker Paravision software (PV6) and *T*_*1*_-maps were generated by a mono-exponential fitting method (Matlab, R2013a, The MathWorks Inc., USA).

## Results

### Characterization of Gd(III)-DOTA-AaLS-R108C

The extent of the Gd(III)-DOTA-conjugation was determined by ESI-TOF MS, following the dissociation of Gd(III)-DOTA-mal treated AaLS-R108C. The subunit molecular masses of AaLS-R108C and Gd(III)-DOTA-mal treated AaLS-R108C were determined to be 27415.0 and 28095.0 Da, respectively, which are in an excellent agreement with the corresponding calculated values of 27413.6 and 28096.9 Da and only one mass peak was observed (Fig. 1B). Mass spectrometric data indicated that all subunits of AaLS-R108C were modified with only one Gd(III)-DOTA complex (60 Gd(III)-DOTA per cage). The Gd-content of Gd(III)−DOTA conjugated AaLS-R108C was independently measured with inductively coupled plasma-mass spectrometry (ICP-MS) and the Gd-content was determined to be 0.86 Gd(III) per subunit, which is slightly lower than the value determined by MS probably due to slight sample loss during ICP-MS sample preparation^23^. To avoid overestimation of the relaxivity of Gd(III)-DOTA–conjugated AaLS-R108C (Gd(III)-DOTA-AaLS-R108C), we used the Gd content as determined by MS^23^. Gd(III)-DOTA-AaLS-R108C was eluted at the same position as untreated AaLS-R108C in size exclusion chromatography (Fig. 1C) and transmission electron microscopic images of negatively stained Gd(III)-DOTA-AaLS-R108C showed its intact cage architecture with the same size as untreated AaLS (~15 nm in diameter, Fig. 1D). Zeta potential measurements revealed that Gd(III)-DOTA-AaLS-R108C had slightly lower negative surface charge (−8.2 mV) than that of AaLS-R108C (−5.5 mV) probably due to the introduced Gd(III)-DOTA. These results demonstrate that Gd(III)-DOTA-mal conjugation to AaLS-R108C does not significantly alter its cage architecture or stoichiometry.

### Relaxivity measurements with phantoms

**Figure 2:**
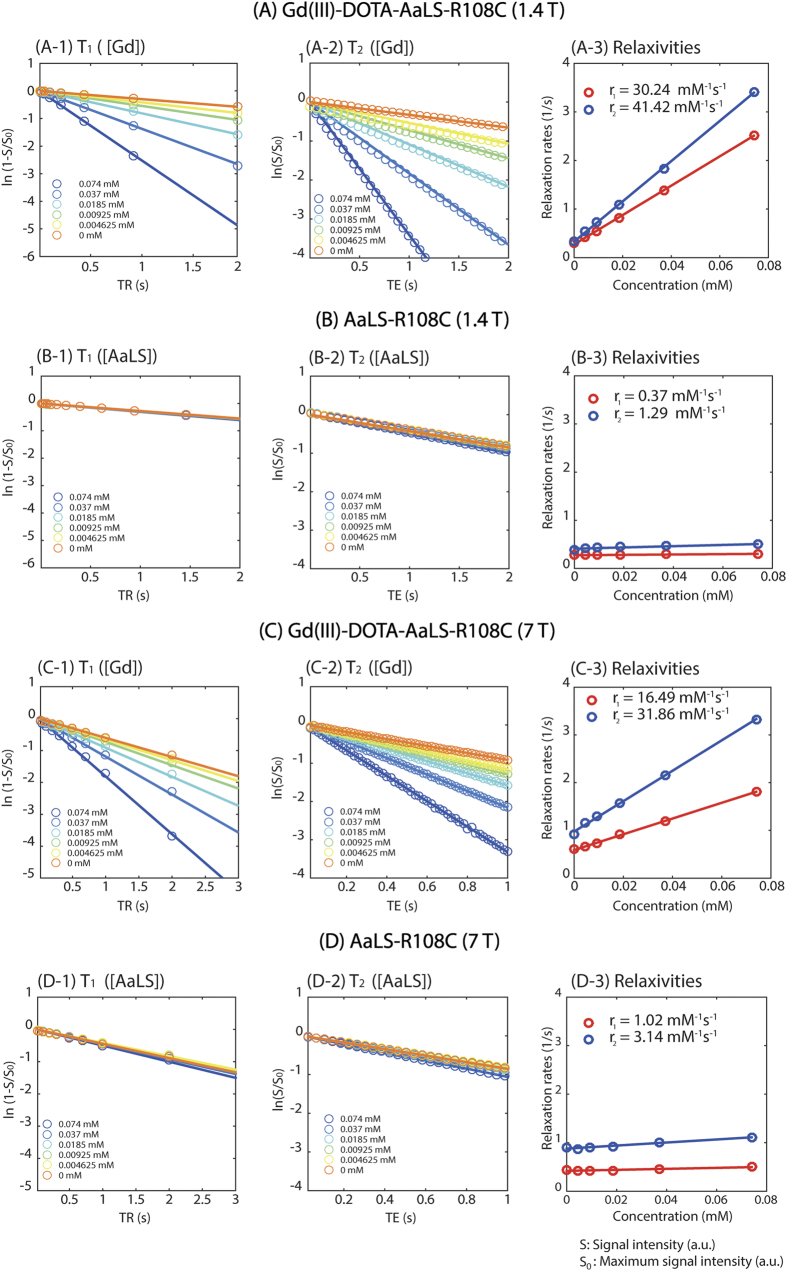
Measurements of *T*_1_ and *T*_2_ relaxation times of Gd(III)-DOTA-AaLS-R108C at 1.4 T (A-1,A-2) and 7 T (C-1,C-2). The inverse values of the *T*_1_ and *T*_2_ relaxation times (*R*_1_ and *R*_2_) are plotted against Gd(III) concentration, whose slopes determine corresponding relaxivities (*r*_1_ and *r*_2_) for 1.4 T (**A-3**) and 7 T (**C-3**). Corresponding relaxation times and relaxivities were measured for untreated AaLS solutions and shown for 1.4 T (**B-1**–**B-3**) and 7 T (**D-1**–**D-3**).

**Figure 3:**
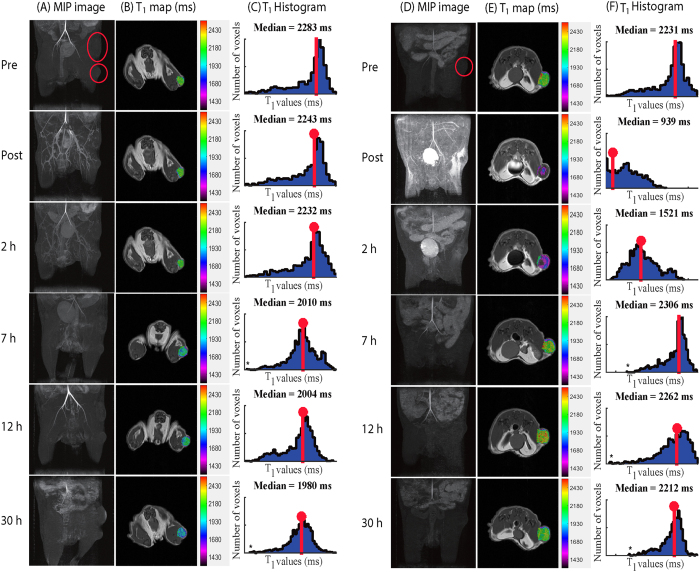
Representative (A) 3D MIP images, (**B**) *T*_1_-maps, and (**C**) Histogram of *T*_1_ values at various time points after injection of Gd(III)-DOTA-AaLS-R108C. Representative (**D**) 3D MIP images, (**E**) *T*_1_-maps, and (**F**) Histogram of *T*_1_ values at various time points after injection of conventional Gd-DOTA.

The *T*_1_ relaxation times became shorter under both 1.4 T and 7 T, as the Gd(III) concentrations increased (Fig. 2A-1) suggesting that the Gd(III)-DOTA-AaLS-R108C supramolecular template accelerated the recovery of net magnetization. Corresponding *T*_2_ relaxation times also decreased as Gd(III) concentrations increased (Fig. 2A-2). Gd(III)-DOTA-AaLS-R108C showed higher *T*_1_ relaxivity (*r*_1_ = 30.24 mM^−1^s^−1^, *r*_2_ = 41.42 mM^−1^s^−1^, Fig. 2A-3) per Gd(III) under 1.4 T (37 °C) than did free Gd(III)-chelating agents (4–5 mM^−1^s^−1^). Although the *T*_1_ relaxivity of Gd(III)-DOTA-AaLS-R108C decreased (*r*_1_ = 16.49 mM^−1^s^−1^, *r*_2_ = 31.86 mM^−1^s^−1^, Fig. 2C-3) under high magnetic field (7 T, room temp.), the measured *r*_1_/*r*_2_ values of Gd(III)-DOTA-AaLS-R108C were 0.73 and 0.52 for 1.4 T and 7 T, respectively, indicating that Gd(III)-DOTA-AaLS-R108C remains as the optimal *T*_1_ contrast agent at 7 T^23^,^26^. The *T*_*1*_ and *T*_*2*_ relaxation times measurements with increasing concentration of untreated AaLS-R108C at identical experimental conditions were shown in Fig. 2B-1 for 1.4 T. Corresponding relaxations times measurements were shown in Fig. 2D-1 for 7 T. The relaxivities of untreated AaLS-R108C at 1.4 T (*r*_1_ = 0.37 mM^−1^s^−1^, *r*_2_ = 1.29 mM^−1^s^−1^, Fig. 2B-3) and 7 T (r_1_ = 1.02 mM^−1^s^−1^, *r*_2_ = 3.14 mM^−1^s^−1^, Fig. 2D-3) were determined as reference.

To evaluate the potential usage of Gd(III)-DOTA-AaLS-R108C as an *in vivo T*_1_ contrast agent at high magnetic field, we captured *T*_1_-weighted MR images of the phantom using a volume coil with a 7 TMRI scanner using a RAREVTR sequence at 4 TR values (100, 500, 1000, and 2000 ms). *T*_1_-weighted *in vitro* phantom images of PBS control (S1A), AaLS-R108C only (S1B) and Gd(III)-DOTA-AaLS-R108C with increasing concentrations (S1C) were shown in supporting Fig. 1. While the MR signal intensities of Gd(III)-DOTA-AaLS-R108C were significantly enhanced at short TRs (500 and 1000 ms), those of the PBS control and AaLS-R108C were not, as shown in supporting Figure S1C, S1A, and S1B, respectively. As the Gd concentration of Gd(III)-DOTA-AaLS-R108C is increased, the accelerated *R*_1_ values of Gd(III)-DOTA-AaLS-R108C led to contrast enhancement resulting in brighter images at short TRs as shown in Figure S1C. For the comparisons with conventional DOTAREM, saturation recovery signals with RAREVTR at multiple TR values were plotted together, where the Gd concentrations (0.0185, 0.037, and 0.074 mM) were kept same for both Gd(III)-DOTA-AaLS-R108C and DOTAREM as shown in S1D. Increased *R*_1_ values were apparent for Gd(III)-DOTA-AaLS-R108C at the same concentration, which led to enhanced *r*_1_ relaxivity over conventional DOTAREM at 7 T.

### *In vivo* characterizations

3D MR angiograms and *T*_1_ maps of tumor-bearing mice were generated to evaluate both the feasibility of MR angiography and the tumor accumulation efficacy using newly developed Gd(III)-DOTA-AaLS-R108C. Identical experiments and analysis were repeated with conventional DOTAREM for direct comparisons. From these MR data, region of interest (ROI) analysis was also employed to measure regionally averaged *T*_1_ values and corresponding signal enhancement.

The decreasing trend in *T*_1_ values of the tumor region of mice was consistently observed after the time point of ~7 h post-Gd(III)-DOTA-AaLS-R108C injection. Figure S2A and S2B illustrates this, showing *T*_1_ maps before and 30 h after the injection. Supplementary Figure S2C shows the corresponding *T*_1_ fitting results at both time points, respectively. Decreased *T*_1_ values in the tumor region could clearly be observed 30 h after the injection of Gd(III)-DOTA-AaLS-R108C both with *T*_1_ maps and corresponding raw *T*_1_ fitting results.

MIP images of tumor bearing mice taken at sequential time points (pre, post, 2 h, 7 h, 12 h, and 30 h) after the injection of Gd(III)-DOTA-AaLS-R108C were plotted in the first column of Fig. 3A. Immediately after the injection, blood vessels were brightened as the *T*_1_ values in the vessels significantly decreased due to the contrast agent injection. As time progressed, positive contrast in blood vessel diminished and ~7 h after the injection, the positive contrast in the tumor region started to appear. *T*_1_ shortening of tumor regions was further confirmed by *T*_1_ maps shown in the second column of Fig. 3B and the median-shift of the *T*_1_ histogram distribution of the whole 3D tumor volume, shown in the third column of Fig. 3C. Identical analysis was performed with conventional DOTAREM as shown in Fig. 3D–F. Blood vessels were also brightened right after the injection and dimming. Positive *T*_1_ contrast in the tumor region was apparent up to 2 h after the injection of DOTAREM, and positive *T*_1_ contrast diminished and the *T*_1_ values were restored to original values 7 h post-injection. Longitudinal maximum intensity projection (MIP) images before and after the injections of Gd(III)-DOTA-AaLS-R108C (*n* = 4) and DOTAREM (*n* = 2) were shown in supporting Figure S3.

**Figure 4:**
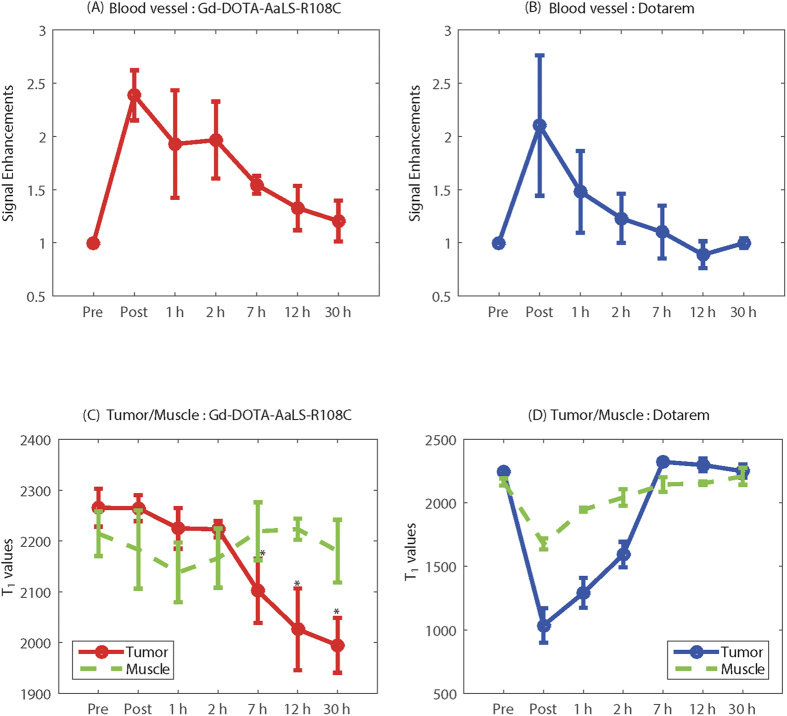
Temporal signal enhancement in arterial region after injection of (**A**) Gd(III)-DOTA-AaLS-R108C and (**B**) Gd-DOTA. (**C**,**D**) Temporal variation of median *T*_1_ values in tumor and muscle regions after the injection of Gd(III)-DOTA-AaLS-R108C and Gd-DOTA, respectively. (* represents statistical significance with p < 0.05).

Figure 4A,B present ROI analysis, showing the temporal trend of signal enhancement measured in the arterial region after injection with Gd(III)-DOTA-AaLS-R108C and conventional DOTAREM. The signal enhancement trend in artery caused by both agents had a similar pattern. Directly after injection, maximal arterial signal enhancements were obtained from both contrast agents, this enhancement diminished as time progressed. A slower decreasing slope of arterial signal enhancement with Gd(III)-DOTA-AaLS-R108C than that from conventional DOTAREM was observed presumably from the elongated intravascular residence time of Gd(III)-DOTA-AaLS-R108C due to its size and hydrophilic surface. No meaningful arterial signal enhancements were observed 7 h and 30 h after injection with DOTAREM and Gd(III)-DOTA-AaLS-R108C, respectively.

Temporal variations of *T*_1_ values in both tumor and muscle on the other side of flank after injection with Gd(III)-DOTA-AaLS-R108C and DOTAREM were presented in Fig. 4C,D. For Gd(III)-DOTA-AaLS-R108C, the *T*_1_ value in the tumor slowly decreased in the first 2 h post-injection, and started to show a statistically significant change from 7 h post-injection (p < 0.05), while *T*_1_ in muscle showed no significant change at any time point. For DOTAREM on the other hand, the *T*_1_ value in the tumor reached the minimum value at the initial time point right after the injection, and then rapidly rebounded and returned to its original level at 7 h. *T*_1_ in muscle showed a similar trend to *T*_1_ in tumor, with relatively reduced changes of *T*_1_ values at each time point.

## Conclusions and Discussions

The high *T*_1_ relaxivity of Gd(III)-DOTA-AaLS-R108C may result from the slow tumbling rate of its large cage architecture^28^. Most previous studies performed with VLPs and protein complexes as supramolecular conjugates haven shown relaxivities of 10–50 mM^−1^s^−1^, depending on experimental conditions^23^,^24^,^25^,^26^,^27^. When compared with experiments of Gd(III)-DOTA chelating agent conjugates we have performed previously, our measured value at 1.4 T (30.24 mM^−1^s^−1^) was slightly lower than that of large VLPs, P22 K118C WB (35.8 mM^−1^s^−1^), but slightly higher than small sized ferritin (19.9 mM^−1^s^−1^)^23^. The *T*_1_ enhancement ability (*r*_1_/*r*_2_) tends to decrease significantly as the magnetic field is increased and often represents a major problem when applying macromolecular contrast agents at high field. The measured *r*_1_/*r*_2_ ratio (0.52) of Gd(III)-DOTA-AaLS-R108C at high magnetic field makes it attractive as a *T*_1_ contrast agent at 7 T. It appears that the molecular hindrance mechanism and resulting dynamics of Gd(III)-chelating agent complexes conjugated to lumazine synthase determine overall relaxation characteristics, but the exact explanation is unresolved at this point. Future relaxation simulation may shed more light on these experimental observations.

*In vivo* MIP images after the injection of Gd(III)-DOTA-AaLS-R108C show clearly defined vasculature, as shown in Fig. 3A. Compared with Gd-DOTA, which exhibited fast leakage in a rodent model of vasculature imaging, intravascular Gd(III)-DOTA-AaLS-R108C appears to provide robust MR angiography at 7 T. The temporal trend of signal enhancement in the arterial region shows that optimal signal enhancement of the vasculature is achieved within 2 h of intravenous injection of Gd(III)-DOTA-AaLS-R108C.

As the longitudinal relaxation rate (*R*_*1*_) in tumor is generally known to be proportional to Gd(III) concentration, the absolute *T*_1_ (*1/R*_*1*_) map of tumor and muscle regions on the other hind leg enables the monitoring of temporal variations in Gd(III) accumulation of both regions, with minimized animal repositioning and slice mismatch errors occurring in longitudinal follow up studies. In other words, decreasing absolute *T*_1_ values in tissue should reflect increasing Gd(III) concentration of corresponding region. The *T*_1_ value of the tumor decreased after the injected Gd(III)-DOTA-AaLS-R108C gradually migrated to the tumor region. A statistically significant decline in *T*_1_ value was observed 7 h post-injection, which coincides with a significant drop in signal enhancement in the arterial region. Compared to the short retention time (<2 h) of DOTAREM, due to its relatively small size and low molecular weight^45^, Gd(III)-DOTA-AaLS-R108C showed a prolonged retention time in tumor. A consistent *T*_1_ value in the other hind leg muscle away from the tumor supported the tumor-specific migration of Gd(III)-DOTA-AaLS-R108C, presumably due to the enhanced permeability and retention effects of the tumor^46^,^51^. These observations could be considered as the results of the abnormal characteristics of tumor tissue. In general, it is known that tumor tissues exhibit leaky vasculature and ineffective lymphatic drainage due to rapid and defective angiogenesis^52^. Hence, the tumor vasculature easily permits adequately sized macromolecules in plasma to escape from the tumor vessels and accumulate in tumor tissue for a specific time^51^. Meanwhile, although the long retention time of Gd(III)-DOTA-AaLS-R108C in tumor could be a strength in applications relating to anti-tumor therapy or drug delivery, it may raise toxicity concerns due to the release of free Gd(III) from prolonged retention, and further *in vivo* toxicity study should be followed.

This study demonstrates that newly developed Gd(III)-DOTA-AaLS-R108C could be successfully applied as a positive *T*_1_ MR contrast agent at high field, and utilized as a high-resolution vasculature imaging agent, active within 2 h of injection. Its prolonged retention time in tumors may be a key advantage for a potential theranostic nanoplatform, as well as for future scientific investigations of optimized MRI *T*_*1*_ contrast agent at higher magnetic field.

## Additional Information

**How to cite this article**: Song, Y.K. *et al.* Lumazine Synthase Protein Nanoparticle-Gd(III)-DOTA Conjugate as a *T*_1_ contrast agent for high-field MRI. *Sci. Rep.* **5**, 15656; doi: 10.1038/srep15656 (2015).

## Supplementary Material

Supplementary Information
