# Supplementary material for: Lumazine Synthase Protein Nanoparticle-Gd(III)-DOTA Conjugate as a T1 contrast agent for high-field MRI
Source: Sci Rep. 2015 Oct 23;5:15656. doi: 10.1038/srep15656 (PMC4616051; doi:10.1038/srep15656)

Supporting Information

**Lumazine Synthase Protein Nanoparticle-Gd(III)-DOTA Conjugate as a *T*_1_ contrast agent for high-field MRI**

*YoungKyu Song^a,1^, Young Ji Kang^b,1^, Hoesu Jung^a,1^, Hansol Kim^b^, Sebyung, Kang^b,*^, and HyungJoon Cho^a,*^*

Figure S1. *T*_1_-weighted *in vitro* phantom images of PBS control (A), AaLS-R108C only (B) and Gd(III)-DOTA-AaLS-R108C with increasing concentrations (C), all at 7 T and obtained using a RAREVTR sequence at 4 TR values (100, 500, 1000, and 2000 ms). For the comparison with conventional DOTAREM, saturation recovery signal with RAREVTR at multiple TR values were plotted together at a few identical Gd concentrations (0.0185, 0.037, and 0.074 mM) of Gd(III)-DOTA-AaLS-R108C and DOTAREM (D). Increased R1 values were apparent for Gd(III)-DOTA-AaLS-R108C.


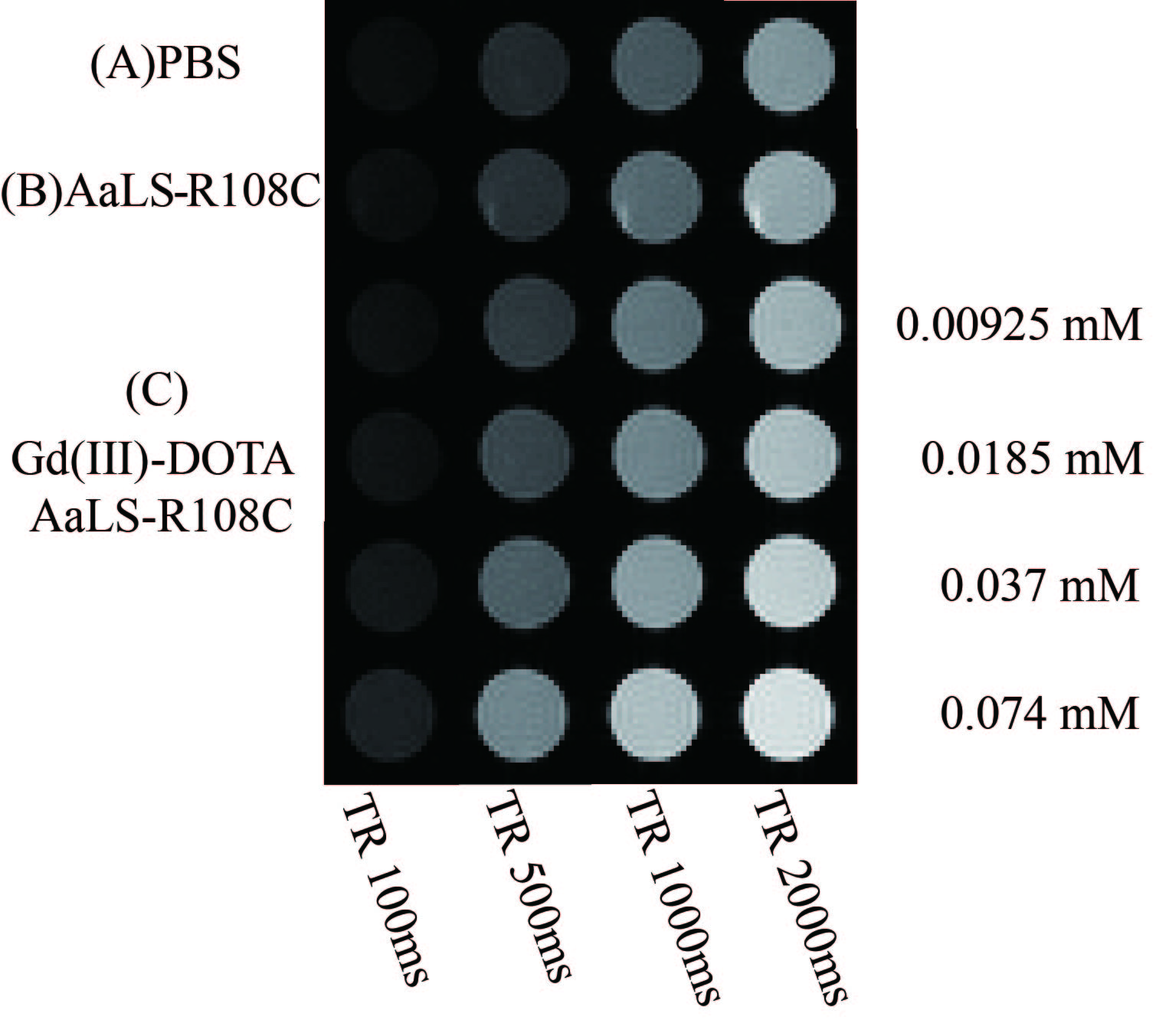


Figure S2. (A,B) Representative *T*_1_ map and (C) *T*_1_ fittings of ROI over the tumor prior to (blue square) and 30 h (red square) post-injection of Gd(III)-DOTA-AaLS-R108C.


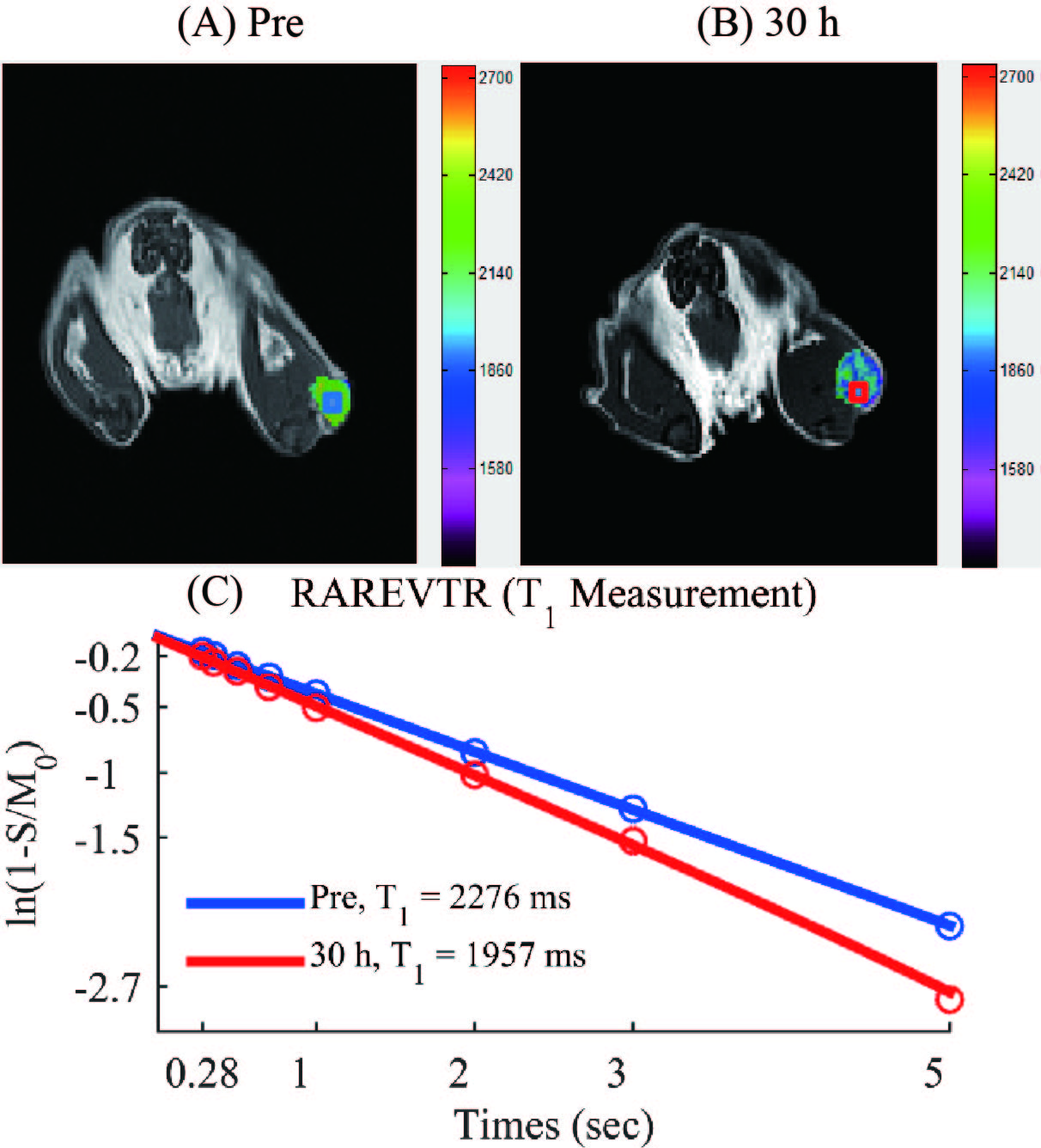


Figure S3. Longitudinal maximum intensity projection (MIP) images before and after injections of Gd(III)-DOTA-AaLS-R108C (*n*=4) and DOTAREM (*n*=2)


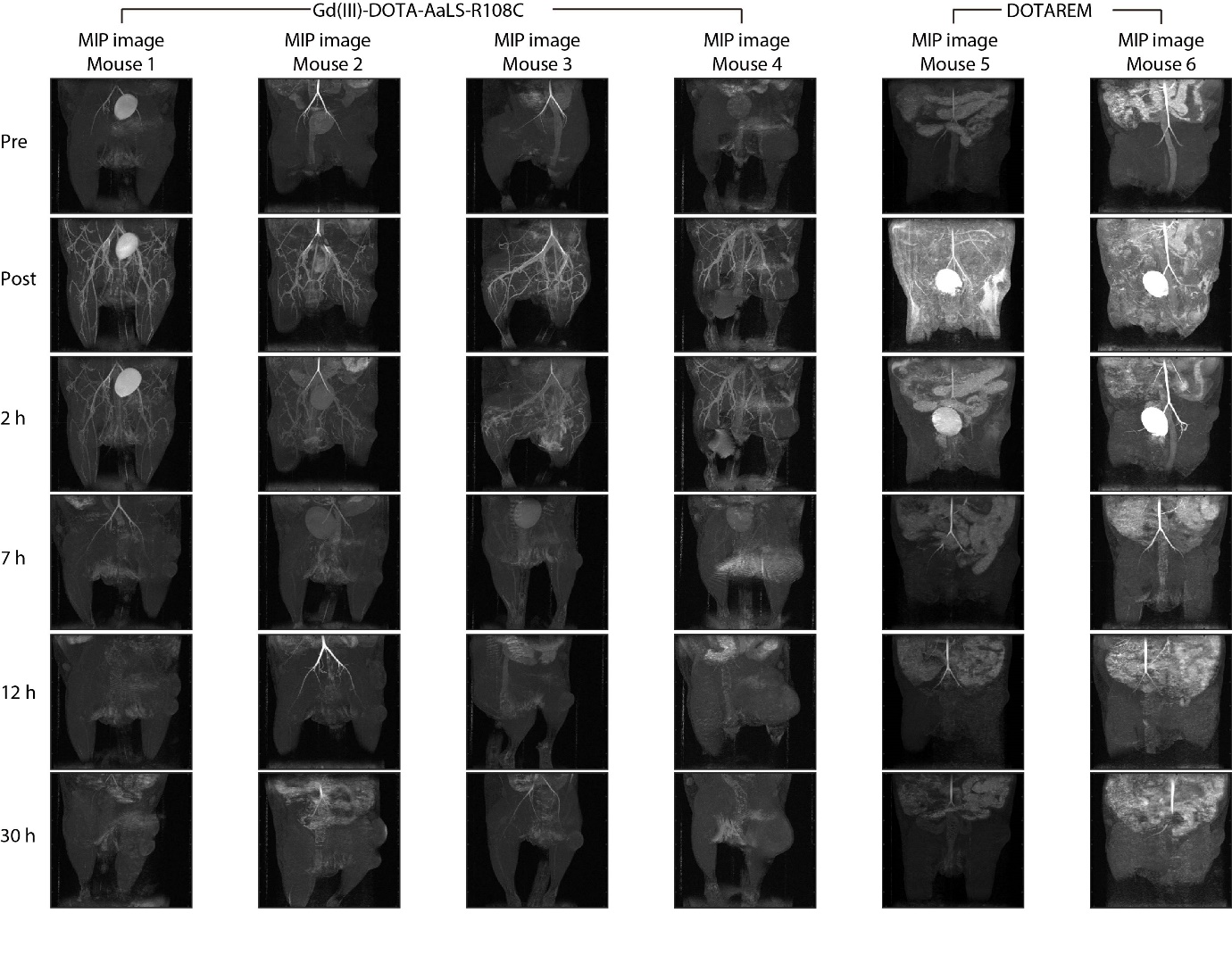

Supplement: Supplementary Information [file srep15656-s1.doc]
